# A clinical study into the impact of brief aerobic exercise on the cardiopulmonary function of patients diagnosed with hyperlipidemia

**DOI:** 10.3389/fcvm.2025.1484693

**Published:** 2025-04-16

**Authors:** Hua Zhang, Ya-Ru Ge, Li Feng, Jing Zhang, Ning Wang, Xiao-Yan Zhang, Cong Liu

**Affiliations:** Community Medical Service Center, Beijing Shijitan Hospital, Beijing, China

**Keywords:** aerobic exercise, cardiopulmonary function, copeptin (CPP), fibroblast, hyperlipemia, growth factor 21 (FGF21)

## Abstract

**Objective:**

The aim of this study was to examine the effectiveness of brief aerobic exercise on patients diagnosed with hyperlipidemia.

**Methods:**

Seventy patients diagnosed with hyperlipidemia were enrolled from community health centers between May 2023 and October 2023. They were randomly assigned into two groups: test and control group, each consisting of 35 patients. The test group received personalized exercise recommendations along with routine medication, while the control group only received routine medication. The effectiveness of the intervention was assessed after 3 months. Various indicators including blood pressure, lipid levels, changes in cardiopulmonary function, 6-minute walk distance (6MWD) test results, the percentage of 6MWD relative to the predicted value (6MWD%Pred), copeptin (CPP), and fibroblast growth factor 21 (FGF21) were compared, before and after the intervention within each group and between the two groups.

**Results:**

After 3 months of personalized exercise, the test group showed significantly higher levels in three indicators compared to the control group. Specifically, the test group exhibited higher metabolic equivalent (MET) values [($\bar{x}$ ± SD*,* 4.6 ± 0.27 METs) vs. ($\bar{x}$ ± SD, 3.8 ± 1.06 METs); *P* < 0.05], longer 6-minute walk distance (6MWD) [($\bar{x}$ ± SD, 488.08 ± 40.59 meters) vs. ($\bar{x}$ ± SD, 366.76 ± 160.49 meters); *P* < 0.05], and a higher percentage of 6MWD relative to the predicted value (6MWD %Pred) [($\bar{x}$ ± SD, 92.8 ± 14.05) vs. ($\bar{x}$ ± SD, 71.3 ± 30.69); *P* < 0.05]. Additionally, within the test group, there was a significant increase in MET, 6MWD, and 6MWD%Pred compared to baseline (*P* < 0.05).

**Conclusion:**

Short-term aerobic exercise shows significant improvement in the cardiopulmonary function of patients with hyperlipidemia. However, it did not exhibit significant efficacy in managing hyperlipidemia.

## 1 Introduction

Hyperlipidemia is characterized by elevated levels of total cholesterol (TC) and triglycerides (TG), often from the result of a diet rich in calories, sugar, and fat (1). Thompson et al. reported the beneficial effects of increased physical activity and exercise on cardiovascular morbidity and mortality (2). A review article concluded that aerobic resistance exercise and the combination of aerobic and resistance training affect cholesterol and blood lipid levels (3). Research has indicated that appropriate aerobic exercise can improve metabolic disorders, particularly among elderly individuals (4). It has been proven that aerobic exercise can improve the lipid profile of blood, improve hyperlipidemia, improve the function of endothelial cells, enhance the function of mitochondrial oxygenation, and delay the progression of atherosclerosis (5, 6). Shujuan Hu et al. found that aerobic exercise ameliorated hyperlipidemia by effectively inhibiting SPARC signaling after 6 weeks of treadmill exercise training in hyperlipidemic rats (7).

Cardiopulmonary function serves as a comprehensive indicator of overall health, reflecting the intricate processes involved in oxygen transport and utilization. There is growing evidence that low levels of coronary flow reserve (CRF) are associated with a higher risk of CVD and higher all-cause mortality (8–10). Importantly, improvements in CRF are associated with a reduced risk of death (11). The level of CRF is influenced by a number of factors including genetics (12). Parameters such as heart function, lung ventilation, and vascular system efficiency play crucial roles in determining cardiopulmonary function. A more practical measure of CRF is the estimated peak metabolic equivalent (1 Mets expressed as 3.5 ml of oxygen consumed per minute per kilogram of body weight), whereas MET can be obtained from a 6-minute walk test. Metabolic equivalent (MET), measured using specialized equipment, is commonly utilized to prescribe aerobic exercise regimens (13, 14). The relatively strong prognostic value of exercise capacity is further emphasized by studies showing that each 1 Mets increase in exercise capacity is associated with a 12% increase in survival (15). A study by the National Sports and Heart Program (16) found that in patients with myocardial infarction (MI), a 1 Mets increase in exercise capacity was associated with a reduction in mortality from any cause, ranging from 14% to 19%.

However, there is a scarcity of studies investigating cardiovascular function assessment using MET and evaluating the impact of aerobic exercise on cardiovascular function in patients with hyperlipidemia. Thus, in this study, we explore the effects of short-term aerobic exercise on cardiovascular function in patients with hyperlipidemia and elucidate potential underlying mechanisms.

## 2 Study material

### 2.1 Study participants

Seventy patients diagnosed with hyperlipidemia and attending the community service centers affiliated with Beijing Shijitan Hospital, Capital Medical University, between May 2023 and October 2023, were enrolled in this study. They were randomly allocated into two groups, the test and control groups, each comprising 35 cases, using a random number table. Inclusion criteria were as follows: (1) Diagnosis of hyperlipidemia, (2) Age ≥45 years old, and (3) Voluntary signing of informed consent forms. Exclusion criteria were: (1) Presence of severe cardiovascular or cerebrovascular disease, lung disease, liver or kidney dysfunction, hematologic disease, or tumor in addition to hyperlipidemia, (2) Severe mental illness, and (3) Mobility dysfunction. The study was approved by the Ethics Committee of Beijing Shijitan Hospital, Capital Medical University (Approval No.: sjtky11-1x-2023 026). Prior to participation, patients received comprehensive information regarding the study's objectives, potential risks, and their responsibilities. They provided informed consent after fully understanding the study and its implications.

### 2.2 Intervention

Both the test and control groups received routine medication, including lipid-lowering and antihypertensive medications, throughout the study. In addition to these medications, the test group was provided with a personalized exercise recommendations. This program was designed according to the principles of the FITT (Frequency, Intensity, Type, Time) model, which tailors the exercise regimen to the individual's needs. Initially, each patient underwent a 6-minute walk test (6MWT) to assess their baseline cardiovascular function, specifically measuring their maximum heart rate (17). The 6MWT is a widely used, low-complexity test for evaluating functional exercise capacity, particularly in patients with moderate-to-severe pulmonary disease. It involves walking as far as possible along a 30 m corridor for 6 min, with the primary outcome being the 6-minute walk distance (6MWD), a predictor of morbidity and mortality in various chronic conditions. Unlike cardiopulmonary exercise stress testing, the 6MWT does not require complex equipment or technical expertise, making it practical for clinical assessments. Based on the 6MWT results, physicians developed personalized exercise prescriptions aimed at achieving moderate aerobic intensity (40%–60% of the patient's maximum heart rate or VO_2_ max). The exercise regimen, consisting primarily of aerobic exercises, was progressively adjusted to ensure that it matched the patient's physical capacity and rehabilitation goals. The exercise program was consistently implemented over a period of 12 weeks.

The Dr. Walker 6-minute walking monitoring and analysis system (WKA202008011, Wocaring Medical Equipment Co., Ltd., China) was used to track real-time data during each 6MWT. This intelligent system provided real-time monitoring of key physiological parameters such as electrocardiogram (ECG), blood pressure, respiratory rate, pulse oxygen saturation, exercise distance, and step count, all of which were used to evaluate the patient's exercise endurance and adjust the exercise prescription as needed.

### 2.3 6MWT

Both groups underwent the 6-minute walk test (6MWT). Prior to the test, participants received comprehensive health training to fully understand the purpose and procedure of the test. The 6MWT was conducted both before and after the intervention. Here's how the test was carried out: Before commencing the test, patients were connected to dynamic electrocardiographic monitoring devices to monitor their heart rate continuously during the test. Participants were then instructed to walk back and forth along a 20 m straight indoor corridor, with colored ribbons marking the turning points at each end. To ensure accurate results, participants fasted for a minimum of two hours before the test and refrained from engaging in vigorous physical activities during this period. They wore comfortable attire and suitable footwear for the test. Participants were encouraged to exert maximum effort during the walk. The total distance covered by each participant within the six-minute duration was meticulously recorded.

### 2.4 Observation indicators

Baseline data included participants’ gender, age, resting systolic blood pressure (SBP), resting diastolic blood pressure (DBP), and heart rate (HR). (2) The indicators assessed during the 6-minute walk test (6MWT) were metabolic equivalent (MET), 6-minute walk distance (6MWD), and the percentage of 6MWD relative to the predicted value (6MWD%Pred). (3) Additional indicators measured using enzyme-linked immunosorbent assay (ELISA) kits included total cholesterol (TC), triglyceride (TG), high-density lipoprotein cholesterol (HDL-C), low-density lipoprotein cholesterol (LDL-C), copeptin (CPP), and fibroblast growth factor 21 (FGF21).

### 2.5 Statistical methods

Data analysis was conducted using SPSS 26.0 statistical software. Normally distributed measurement data were presented as mean ± standard deviation (x̅ ± s), while non-normally distributed measurement data were presented as median and interquartile range [median (IQR)]. Enumeration data were expressed as frequency and composition ratio [*n* (%)]. Independent samples t-test or paired samples t-test was applied for normally distributed and homoscedastic measurement data, while non-parametric tests were used for data that did not meet these assumptions. Enumeration data were analyzed using Fisher's exact test. Statistical significance was set at a *P*-value less than 0.05 (*P* < 0.05).

## 3 Study results

### 3.1 Baseline data

There were totally 70 participants in this study, comprising 32 males (46%) and 38 females (54%). Within the test group, there were 14 males (40%) and 21 females (60%), while in the control group, there were 18 males (54%) and 17 females (46%). The average age of participants in the test and control groups was 61 ± 8.7 and 60 ± 9.8 years, respectively. At baseline, there were no statistically significant differences between the test and control groups in SBP, DBP, MET, target heart rate, 6MWD, 6MWD%Pred, TC, TG, LDL-C, and FGF21 (*P* > 0.05), indicating comparability between the two groups. However, the test group exhibited significantly higher levels of HDL-C and CPP compared to the control group (*P* < 0.05), as shown in Table 1.

**Table 1:** Comparison of baseline data between the two groups.

Variables at baseline	Test group (*n* = 35)	Control group (*n* = 35)	Test results	*P* value
Age, median (IQR), years	63 (56,68)	65 (55,67)	Z = −0.129	0.897
Gender, *n* (%)			*χ*^2^ = 0.921	0.337
Male	14 (53%)	18 (59%)		
Female	21 (47%)	17 (41%)		
SBP (mean ± SD, mmHg)	132 ± 14	135 ± 14	t = −0.572	0.569
DBP (mean ± SD, mmHg)	76 ± 8	76 ± 10	t = −1.003	0.319
TC (mmol/L)	3.91 ± 0.77	3.74 ± 0.67	t = −0.563	0.575
TG (mmol/L)	2.22 (1.50,3.07)	2.40 (1.76,3.20)	Z = −0.559	0.576
HDL-C (mmol/L)	1.35 (1.13,1.90)	1.24 (1.76,3.20)	Z = −2.058	0.040*
LDL-C (mmol/L)	2.49 ± 0.59	2.51 ± 0.76	t = −0.823	0.414
METs (mean ± SD)	4.4 ± 0.4	4.2 ± 0.5	t = 1.494	0.141
Heartbeat [median (IQR), beat/minute]	124 (120,130)	123 (120,130)	Z = −0.465	0.642
6MWD (m)	458.59 ± 55.14	439.84 ± 77.80	t = 1.551	0.127
6MWD%Pred (%)	84 ± 11	82 ± 14	t = 1.461	0.150
CPP (pmmol/L)	202.02 ± 130.95	121.57 ± 51.22	t = 2.050	0.047*
FGF21 (nM)	4.87 ± 0.73	4.57 ± 0.74	t = 1.160	0.253

Normally distributed data are presented as mean ± standard deviation (x̅ ± s), while non-normally distributed data are represented by median (interquartile range, IQR). Categorical data are expressed as counts (percentages). *indicates statistical significance at *P* < 0.05.

### 3.2 Indicator comparison between the two groups after three months of intervention

After three months of intervention with personalized exercise, follow-up assessments were conducted on all study participants. In the test group, 33 individuals underwent blood sampling and 6MWT, resulting in a participation rate of 94%. In the control group, 24 individuals underwent blood sampling and 6MWT, with a participation rate of 69%. Comparing the results revealed that the test group showed significantly higher average levels of MET, 6MWD, and 6MWD%Pred compared to the control group after the intervention, with the following values, respectively: (4.6 ± 0.27 METs) vs. (3.8 ± 1.06 METs) (*P* < 0.05), (x̅ ± s, 488.08 ± 40.59 meters) vs. (x̅ ± s, 366.76 ± 160.49 meters) (*P* < 0.05), and (x̅ ± s, 92.8 ± 14.05%) vs. (x̅ ± s, 71.3 ± 30.69%) (*P* < 0.05). However, there were no statistically significant differences between the test and control groups in SBP, DBP, TC, TG, HDL, LDL, CPP, and FGF21 levels (*P* > 0.05), as illustrated in Table 2.

**Table 2:** Analysis of indicators in the two groups after intervention.

Variables at baseline	Test group (*n* = 35)	Control group (*n* = 35)	Test results	*P* value
SBP (mean ± SD, mmHg)	130 ± 12	132 ± 8	t = −0.684	0.500
DBP (mean ± SD, mmHg)	73 ± 7	72 ± 8	t = 0.402	0.691
TC (mmol/L)	4.00 ± 0.85	4.22 ± 0.82	t = −0.093	0.926
TG (mmol/L)	2.15 ± 0.87	1.96 ± 0.85	t = 0.721	0.474
HDL-C (mmol/L)	1.46 ± 0.35	1.39 ± 0.11	t = 1.850	0.070
LDL-C (mmol/L)	2.31 ± 0.78	2.47 ± 0.79	t = −0.587	0.401
METs (mean ± SD)	4.6 (4.5,4.8)	4.1 (3.6,4.6)	Z = −2.572	0.010*
Heartbeat [median (IQR), beat/minute]	123 (119,126)	122 (120,124)	Z = −0.261	0.794
6MWD (m)	485.3 (471.1,510.5)	401.9 (335.8,475.4)	Z = −2.558	0.011*
6MWD%Pred (%)	94 (84.5,103)	80 (69,83.5)	Z = −2.287	0.022*
CPP (pmmol/L)	185.67 ± 97.76	135.84 ± 43.00	t = 0.913	0.420
FGF21 (nM)	1.58 (1.06,2.88)	1.56 (0.93,2.11)	Z = −0.025	0.980

Normally distributed data are expressed as mean ± standard deviation (x̅ ± s), whereas non-normally distributed data are presented as median (interquartile range, IQR). *indicates statistical significance at *P* < 0.05.

### 3.3 Intra-group comparisons

Within the test group, comparisons were made before and after intervention. Table 3 displays the findings regarding indicator changes in the test group. After the intervention period, participants in the test group showed significantly elevated levels of MET, 6MWD, and 6MWD%Pred (*P* < 0.05). However, no statistically significant differences were observed in SBP, DBP, TC, TG, HDL, LDL, CPP, and FGF21 (*P* > 0.05). Table 4 presents the results of comparisons within the control group between baseline and after three months of intervention. Notably, the control group demonstrated a significant decrease in FGF21 levels following the three-month intervention period.

**Table 3:** Comparison of indicators before and after intervention within the test group.

Variables at baseline	Data at baseline (*n* = 35)	Data after intervention (*n* = 33)	Test results	*P* value
SBP (mean ± SD, mmHg)	132 ± 14	130 ± 12	t = 1.301	0.199
DBP (mean ± SD, mmHg)	76 ± 8	73 ± 7	t = 1.377	0.174
TC (mmol/L)	3.91 ± 0.77	4.00 ± 0.85	t = −0.045	0.964
TG (mmol/L)	2.22 (1.50,3.07)	2.15 ± 0.87	Z = 0.721	0.474
HDL-C (mmol/L)	1.35 (1.13,1.90)	1.46 ± 0.35	Z = 1.850	0.070
LDL-C (mmol/L)	2.49 ± 0.59	2.31 ± 0.78	t = 1.533	0.130
METs (mean ± SD)	4.4 ± 0.4	4.6 (4.5,4.8)	Z = −2.321	0.020*
Heartbeat [median (IQR), beat/minute]	124 (120,130)	123 (119,126)	Z = −0.261	0.794
6MWD (m)	458.59 ± 55.14	485.3 (471.1,510.5)	Z = −2.558	0.011*
6MWD%Pred (%)	84 ± 11	94 (84.5,103)	Z = −2.942	0.003*
CPP (pmmol/L)	202.02 ± 130.95	185.67 ± 97.76	t = 0.603	0.549
FGF21 (nM)	4.87 ± 0.73	1.58 (1.06,2.88)	Z = −0.025	0.980

Normally distributed data are presented as mean ± standard deviation (x̅ ± s), while non-normally distributed data are presented as median (interquartile range, IQR). *P < 0.05 indicates statistical significance.

**Table 4:** Comparison of indicators before and after intervention within the control group.

Variables at baseline	Data at baseline (*n* = 35)	Data after intervention (*n* = 24)	Test results	*P* value
SBP (mean ± SD, mmHg)	135 ± 14	132 ± 8	t = 0.752	0.456
DBP (mean ± SD, mmHg)	76 ± 10	72 ± 8	t = 1.961	0.057
TC (mmol/L)	3.74 ± 0.67	4.22 ± 0.82	t = 0.415	0.679
TG (mmol/L)	2.40 (1.76,3.20)	1.96 ± 0.85	Z = −1.813	0.070
HDL-C (mmol/L)	1.24 (1.76,3.20)	1.39 ± 0.11	Z = −1.990	0.047
LDL-C (mmol/L)	2.51 ± 0.76	2.47 ± 0.79	t = 1.092	0.280
METs (mean ± SD)	4.2 ± 0.5	4.1 (3.6,4.6)	Z = −0.588	0.556
Heartbeat [median (IQR), beat/minute]	123 (120,130)	122 (120,124)	Z = −0.759	0.448
6MWD (m)	439.84 ± 77.80	401.9 (335.8,475.4)	Z = −0.495	0.621
6MWD%Pred (%)	82 ± 14	80 (69,83.5)	Z = −0.283	0.777
CPP (pmmol/L)	121.57 ± 51.22	135.84 ± 43.00	t = −0.639	0.528
FGF21 (nM)	4.57 ± 0.74	1.56 (0.93,2.11)	Z = −4.116	<0.001*

Note: Normally distributed data are represented by x̅ ± s, while non-normally distributed data are represented by median (IQR). *P < 0.05.

## 4 Discussion

Hyperlipidemia prevalently affects the middle-aged and elderly populations and is identified as a contributing factor to coronary heart disease (CHD). Statin medications, despite their clinical utility, manage to curtail cardiovascular events by approximately 40% only (18). Even with the prevalent application of diverse lipid-lowering agents, the occurrence and mortality rates linked to atherosclerosis continue to be substantial (19). Research has illustrated that integrating statin therapy with physical exercise can mitigate lipid abnormalities in the elderly, enhancing cardiovascular health and diminishing the risk of cardiovascular diseases (20). The 6-minute walk test (6MWT) is straightforward and feasible. It serves a dual purpose: evaluating treatment outcomes and cardiac function, as well as indicating outcomes in patients with cardiovascular conditions. This test accurately gauges both submaximal and maximal exercise capacities, thereby reflecting the cardiopulmonary status of patients (21).

Following a three-month intervention involving personalized exercise, the test group exhibited significant improvements in MET, 6MWD, and 6MWD%Pred compared to both their baseline levels (*P* < 0.05) and the corresponding indicators in the control group (*P* < 0.05). In their study, Huili et al. examined the impact of various aerobic exercises on the elderly population with hyperlipidemia and coronary heart disease, finding that high-intensity aerobic exercise was more effective in enhancing cardiovascular function (22). Nonetheless, their investigation did not encompass MET evaluations. As a result of this study, it was confirmed that while there were significant benefits of aerobic exercise on cardiovascular health in patients with hyperlipemia, no substantial enhancements were detected in TC, TG, HDL-C, and LDL-C levels. This outcome might be attributed to the limited size of the sample.

In our study, neither CPP nor FGF21 levels showed significant improvement post-intervention (*P* > 0.05). CPP, a marker substituting for the homologous precursor of arginine vasopressin, is closely linked with metabolic conditions such as diabetes mellitus, hyperlipidemia, and hypertension (23, 24). Research suggests that exercise can markedly elevate circulating CPP concentrations within minutes, and can be attributed to factors like prolonged exercise durations, heightened exercise intensities, and changes in plasma osmotic pressure and serum sodium levels during vigorous physical activity (25–27).

However, the impact of exercise on CPP levels in hyperlipidemia patients remains underexplored. While studies indicate a temporary elevation in CPP levels immediately after intense exercise, followed by a decrease (28), our participants, who had fasted before morning blood collection for CPP level assessment using ELISA kits, showed a reduction in serum CPP levels, though it was not statistically significant. Factors such as exercise intensity and duration, the timing of blood sample collection, and a limited sample size could explain these findings. The FGF21 hormone involved in regulating energy and glucose-lipid metabolism, predominantly expressed in the liver, adipose tissue, and skeletal muscle, is known to be modulated by exercise, offering potential benefits for individuals with metabolic syndromes (29). In rodents and non-human primates, FGF21 treatment has beneficial effects on cardiometabolic outcomes, such as reduction in fat mass and alleviation of hyperglycaemia, insulin resistance, dyslipidaemia, and cardiovascular diseases (30). Furthermore, FGF21 has been implicated in protecting against Alzheimer's disease (31), as well as improving lifespan (32). And studies have shown that exercise can improve metabolic diseases by regulating the expression of FGF21 (33, 34). Despite observing a decrease in serum FGF21 levels among participants after three months, the change was not statistically significant (*P* > 0.05). Taniguchi et al. reported a significant reduction in liver fat and serum FGF21 levels following five weeks of endurance exercise in elderly Japanese men (35). Conversely, Fatemeh et al. observed that 12 weeks of resistance exercise lowered circulating FGF21 levels in elderly men, with or without type 2 diabetes mellitus (36). Interestingly, some research indicates that physical activity can increase FGF21 levels; Daniel et al. found elevated serum FGF21 levels in sedentary women after two weeks of exercise (37). A systematic review highlighted the variable impact of exercise on serum FGF21 levels, noting acute physical activity tends to raise FGF21 levels, whereas long-term exercise (≥4 weeks) either decreases or does not significantly alter FGF21 levels (38).

One important limitation of this study is the relatively short duration of the aerobic exercise regimen. The three-month intervention period may not have been long enough to elicit significant changes in blood lipid levels, as longer and more sustained exercise programs may be required to impact lipid metabolism. Additionally, the intensity and type of exercise may have been insufficient to produce measurable improvements in metabolic markers such as FGF21 and CPP. Future studies with extended intervention periods and varying exercise intensities could help determine the optimal conditions for improving lipid profiles and biomarkers in patients with hyperlipidemia.

## 5 Conclusion

Short-term aerobic exercise led to a significant enhancement in the cardiopulmonary function of patients with hyperlipidemia. However, there was no significant improvement observed in blood lipid levels.

## Data Availability

The original contributions presented in the study are included in the article/Supplementary Material, further inquiries can be directed to the corresponding authors.
